# Chronic kidney disease is potentially an independent prognostic factor for death in Stevens-Johnson syndrome and toxic epidermal necrolysis patients

**DOI:** 10.3389/fmed.2022.939210

**Published:** 2022-08-25

**Authors:** Ploysyne Rattanakaemakorn, Pasita Palakornkitti, Prinpat Pinyowiwat, Phatphitcha Jedee, Kunlawat Thadanipon

**Affiliations:** ^1^Division of Dermatology, Department of Medicine, Faculty of Medicine Ramathibodi Hopital, Mahidol University, Bangkok, Thailand; ^2^Department of Clinical Epidemiology and Biostatistics, Faculty of Medicine Ramathibodi Hospital, Mahidol University, Bangkok, Thailand

**Keywords:** chronic kidney disease, mortality, prognosis, Stevens-Johnson syndrome, survival, toxic epidermal necrolysis

## Abstract

Stevens-Johnson syndrome (SJS) and toxic epidermal necrolysis (TEN) are mucocutaneous conditions associated with high mortality and morbidity. Although several prognostic factors have been proposed, some may have yet to be identified. A 14-year retrospective cohort study of patients with SJS/TEN was conducted at a university-based hospital in Bangkok, Thailand, to explore additional prognostic factors for mortality of patients with SJS/TEN. Medical records of all patients aged ≥18 years who were diagnosed with SJS, SJS-TEN overlap, or TEN between 2007 and 2020 were reviewed. Univariate and multivariate analyses were performed to examine associations between death and potential prognostic factors. A total of 76 patients with a mean age of 52 years were enrolled. Among them, 46, 15, and 15 patients were diagnosed with SJS, SJS-TEN overlap, and TEN, respectively. Overall, 10 patients deceased, marking a mortality rate of 13.2%. Based on an algorithm for assessment of drug causality for epidermal necrolysis, drug was the major cause of disease (96.1%). Allopurinol and trimethoprim/sulfamethoxazole were the most frequent culprit drugs. Univariate analysis revealed nine prognostic factors related to death, i.e., age, malignancy, chronic kidney disease (CKD), coronary artery disease, heart rate >120 beats/min, diagnoses of SJS-TEN overlap and TEN, blood urea nitrogen (BUN) >10 mmol/L, hemoglobin <10 g/dL, and serum albumin <2 g/dL. Causality with regard to drug, drug notoriety, time interval from drug intake to onset of reaction, and timing of culprit drug withdrawal were not significantly associated with death. Four independent prognostic factors for mortality were identified from multivariate analysis, i.e., TEN (risk ratio [RR] 8.29, 95% confidence interval [CI]: 2.71–25.38), malignancy (RR 3.34, 95% CI: 1.68–6.69), BUN >10 mmol/L (RR 3.02, 95% CI: 1.28–7.14), and early-stage CKD (RR 4.81, 95% CI: 2.49–9.28). Our findings suggest that CKD is an independent prognostic factor for mortality of patients with SJS/TEN besides those from the SCORTEN.

## Introduction

Stevens-Johnson syndrome (SJS) and toxic epidermal necrolysis (TEN) are life-threatening mucocutaneous reactions with reported incidence rates of 0.93 to 9.2 per million annually (1–3). In spite of their rarity, reported mortality rates among the patients were high, especially in those with TEN. Previous studies revealed mortality rates of 4.8–5.7% for SJS, 19.4% for SJS-TEN overlap, and 14.8–35.0% for TEN (1, 2, 4).

Drugs are identified as the main cause of disease, while other causes, including *Mycoplasma* pneumonia and viral infections, may account for up to 15% of the cases (5–9). As the causative drug could not be identified in approximately 30% of the patients, the Registry of Severe Cutaneous Adverse Reaction (RegiSCAR) group has consequently developed an algorithm for assessment of drug causality for epidermal necrolysis (ALDEN) which is a practical and reliable tool for identifying the culprit drug for epidermal necrolysis (9–11).

In regard to the prognosis of patients with SJS/TEN, the Severity-of-Illness Score for Toxic Epidermal Necrolysis (SCORTEN) is a tool developed to predict mortality and help clinicians assess disease severity of the patients. The score consists of seven independent prognostic factors, i.e., age >40 years, malignancy, heart rate >120 per min, initial percentage of epidermal detachment >10%, serum urea >10 mmol/L, serum glucose >14 mmol/L, and serum bicarbonate <20 mmol/L (12). A systematic review and meta-analysis demonstrated good accuracy of the SCORTEN in predicting mortality; nevertheless, underestimated and overestimated mortality rates were found for SCORTEN values of ≤ 3 and >3, respectively. The influence of comorbidities, involved body surface area, and patient's age were the major contributors to the misestimation (13). Subsequently, a number of studies have been conducted to identify other prognostic factors (2, 14, 15). However, there is limited evidence on the prognostic potential of several other factors, such as the causality of SJS or TEN, drug notoriety, time interval from drug intake to onset of reaction, and several comorbidities and laboratory findings. Therefore, the present study aimed to identify additional prognostic factors for mortality in patients with SJS/TEN, and to determine the predictive ability of those previously established ones in our setting.

## Materials and methods

### Study design and ethics consideration

A 14-year retrospective cohort study of patients with SJS/TEN was conducted. The study was approved by the Institutional Review Boards of Mahidol University (COA. MURA2017/698 Ref.188) which are in full compliance with International Guidelines for Human Research Protection such as the Declaration of Helsinki.

### Patient selection process

Medical records of all inpatients aged 18 years or over who were admitted to Ramathibodi Hospital, Bangkok, Thailand from January 1, 2007, to December 31, 2020 with an International Classification of Diseases, 10th Revision (ICD-10) code of L51.1 (bullous erythema multiforme), L51.2 (toxic epidermal necrolysis [Lyell]), or L51.8 (other erythema multiforme) were reviewed. Patients with a length of stay of <3 days were excluded to improve the accuracy of the diagnosis (2, 16), but those who died within 3 days of hospitalization were included. Patients who were not hospitalized at Ramathibodi Hospital for the entire course of treatment were also excluded. Other reasons for exclusion were diagnostic codes as a consequence of previous SJS/TEN and incomplete medical record.

The final inclusion of cases was determined by two board-certified dermatologists using the diagnostic criteria proposed by Bastuji-Garin et al. (17). The judgement was based on information from the medical records which included clinical, laboratory, and histopathological findings. The outcome of interest was the survival status at the date of discharge. All causes of death related to the present course of SJS/TEN were considered.

### Identification of cause and culprit drugs

The ALDEN score was applied to assess the causality of SJS/TEN as to whether it was caused by drug (10). Cases exposed to a drug with an ALDEN score of ≥2 were considered to have drug-related SJS/TEN, and those with an ALDEN score of <2 to have non-drug related SJS/TEN. According to the ALDEN algorithm and previous studies, the index day was defined as the date of onset of the symptoms or signs that progressed within 3 days to definite erosions or blisters of the skin or mucous membranes (10, 18–20). The drug with the highest ALDEN score was regarded as the culprit drug. In the event of multiple drugs with equal scores, the case was identified as having multiple culprit drugs. To determine the notoriety of the culprit drug, the classification from the RegiSCAR study with regard to SJS/TEN was used (18, 21).

In patients having drug-related SJS/TEN with a single culprit drug, three more variables were further considered, i.e., time interval form drug intake to onset of reaction, timing of culprit drug withdrawal, and drug notoriety. The index day was used for calculating time interval form drug intake to onset of reaction. Timing of culprit drug withdrawal was classified into early and late drug withdrawal following the determination from a previous study (14). Late drug withdrawal was defined as discontinuing the drug later than the day when a definite sign of SJS/TEN occurred.

### Data collection

Data of patients' characteristics, medical comorbidities, clinical presentations, laboratory findings, treatments, and outcome (i.e., survival status) were collected from the medical records. Previous chronic medical conditions present on admission were regarded as medical comorbidities. Among them, chronic kidney disease (CKD) was defined according to the 2012 KDIGO guideline as abnormalities of kidney structure or function, present for >3 months, with implications for health. The categories of glomerular filtration rate (GFR) comprising of G1 (i.e., GFR ≥90 mL/min per 1.73 m^2^), G2 (i.e., GFR 60–89 mL/min per 1.73 m^2^), G3 (i.e., GFR 30−59 mL/min per 1.73 m^2^), G4 (i.e., GFR 15–29 mL/min per 1.73 m^2^), and G5 (i.e., GFR <15 mL/min per 1.73 m^2^) (22) were adopted in the present study. However, due to the sparsity of data from CKD patients, the categories were combined into G2-G3 (i.e., GFR 30–89 mL/min per 1.73 m^2^ which represents early-stage CKD) and G4-G5 (i.e., GFR <30 mL/min per 1.73 m^2^ which represents late-stage CKD) groups to improve the power of subsequent statistical analysis.

The findings from physical examination including body temperature, heart rate (HR), and final diagnosis of epidermal necrolysis were considered. Regarding the final diagnosis of epidermal necrolysis, the extent of involved body surface area was determined based on the maximum percentage of epidermal detachment during the course of disease and was categorized as SJS, SJS-TEN overlap, and TEN following the disease definition proposed by Bastuji-Garin et al. (17).

The laboratory data considered in the present study include blood urea nitrogen (BUN), hemoglobin level (Hb), white blood cell (WBC) count, platelet count, and serum levels of glucose, bicarbonate (HCO_3_), sodium (Na), potassium (K), aspartate aminotransferase (AST), alanine aminotransferase (ALT), total bilirubin, and albumin. The cut-off levels for dichotomizing HR, BUN, serum glucose, and serum HCO_3_ levels were those used in the SCORTEN. Abnormalities of WBC count, platelet count, serum Na, serum K, serum AST, serum ALT, and serum total bilirubin levels were also determined following those of the SCORTEN study (12). Serum Hb <10 g/dL was classified as low Hb in accordance with a previous study on SJS/TEN by Kannenberg et al. (23). Meanwhile, serum albumin <2 g/dL has been shown to significantly increase the mortality risk in burn patients according to a previous study (24); this cut-off level was therefore applied in the present study.

Data of physical examination and laboratory findings were extracted from the first 5 days of hospitalization, since Guégan et al. (25) reported an excellent performance of the SCORTEN during these first 5 days, as shown by the area under the receiver operating characteristic curve of >0.8. As the highest accuracy of the SCORTEN was found on day 3 of hospitalization, data from day 3 were used when available.

### Statistical analysis

Frequency and percentage were used for describing categorical data. For continuous data, mean and standard deviation (SD) or median and interquartile range (IQR) were applied depending on the distribution of the data. Univariate analysis was performed on all interested variables by simple logistic regression with death as the outcome. The variables with *p*-values <0.2 from univariate analysis were included in a forward selection process, with a forced adjustment for treatments with systemic corticosteroids and intravenous immunoglobulin, to yield a parsimonious logistic regression model used in the multivariate analysis. The resultant odds ratios (OR) and their delta-method standard errors were used to estimate risk ratios (RR) and their 95% confidence intervals (CI) (26), in order to avoid the overestimation of relative risk inherent to ORs in cohort studies such as the present one (27). *P*-values of < 0.05 were considered statistically significant. The Stata statistical software package version 15.0 (StataCorp, College Station, TX) was used in all analyses.

## Results

**Figure 1:**
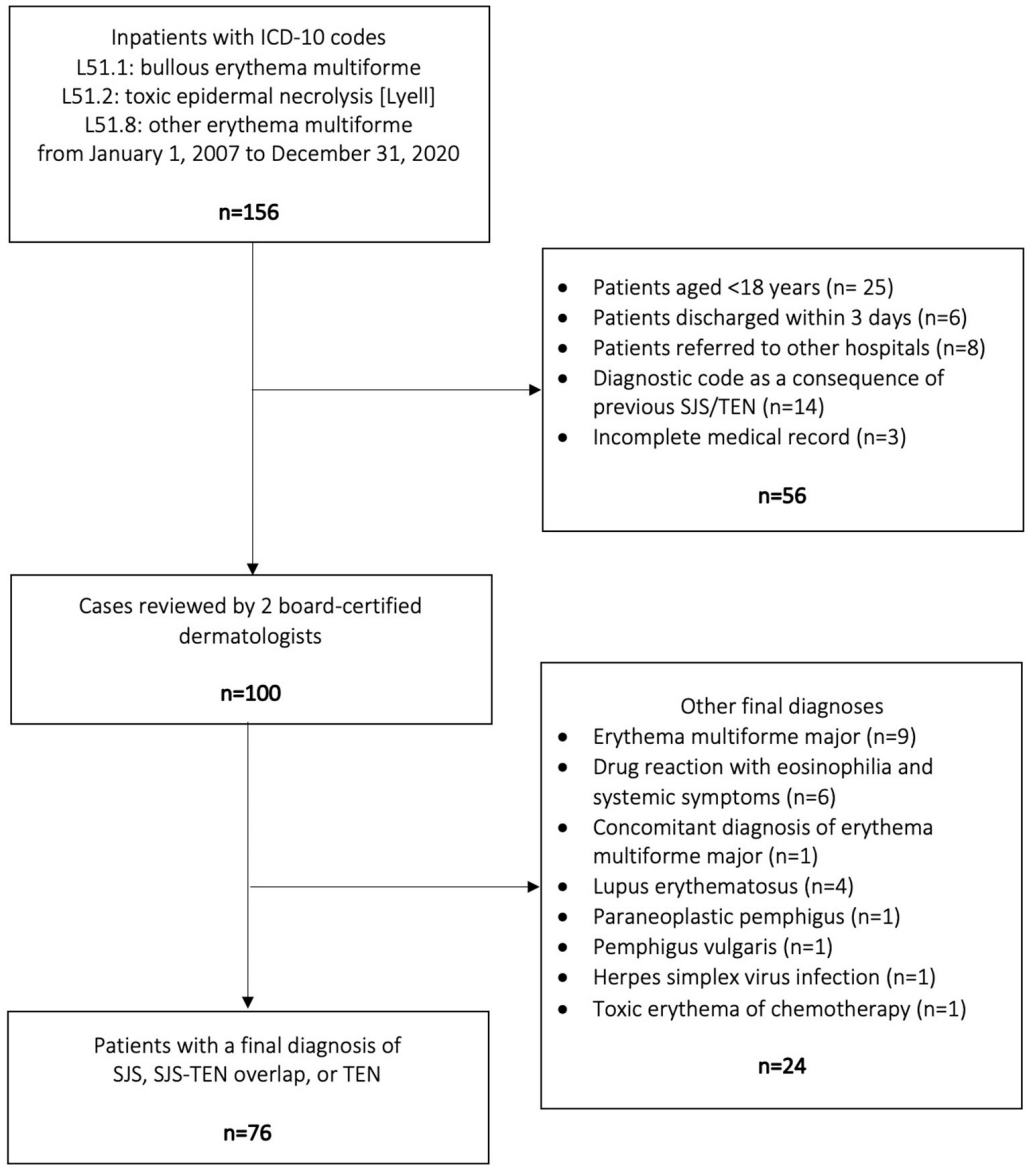
Flow diagram of the patient selection process. SJS, Stevens-Johnson syndrome; TEN, toxic epidermal necrolysis.

There were 156 inpatients assigned with an ICD-10 code of L51.1, L51.2, or L51.8. Among them, 56 were excluded by the reasons displayed in Figure 1. The remaining 100 patients were reviewed by two board-certified dermatologists to exclude other diagnoses than SJS, SJS-TEN overlap, and TEN. Finally, 76 patients were included in the present study (Figure 1).

Overall, the mean (SD) age was 52.0 (18.1) years, and 40 (52.6%) of the patients were female. Of the 76 eligible patients, 46 (60.5%) were diagnosed with SJS, 15 (19.7%) with SJS-TEN overlap, and 15 (19.7%) with TEN. Ten patients died during the course of the disease, resulting in a mortality rate of 13.2% (95% CI: 6.5–22.9%). Among these 10 deceased patients, one had SJS, two had SJS-TEN overlap, and seven had TEN. The mortality rates (95% CI) for SJS, SJS-TEN overlap, and TEN could be estimated at 2.2% (0.06–11.5%), 13.3% (1.7–40.5%), and 46.7% (21.3–73.4%), respectively. The patients' characteristics are summarized in Table 1.

**Table 1:** Patients' characteristics in relation to the death outcome.

	**Total** (*n* = 76)*	**Survivors** **(*n* = 66)**	**Deceased** **(*n* = 10)**
Age, years; mean (SD)	52.0 (18.1)	49.5 (17.5)	68.9 (12.3)
Female; *n* (%)	40 (52.6)	36 (54.6)	4 (40.0)
BMI; mean (SD)^†^	22.2 (4.4)	22.5 (4.4)	19.1 (2.8)
Medical comorbidity; *n* (%)			
Malignancy	12 (15.8)	7 (10.6)	5 (50.0)
Chronic kidney disease	9 (11.8)	5 (7.6)	4 (40.0)
GFR 30–89 mL/min per 1.73 m^2^	4 (5.3)	2 (3.0)	2 (20.0)
GFR <30 mL/min per 1.73 m^2^	5 (6.6)	3 (4.6)	2 (20.0)
Coronary artery disease	4 (5.3)	2 (3.03)	2 (20.0)
Stroke	7 (9.21)	5 (7.6)	2 (20.0)
Hypertension	24 (31.6)	20 (30.3)	4 (40.0)
Diabetes mellitus	13 (17.1)	11 (16.7)	2 (20.0)
Gout	7 (9.2)	6 (9.1)	1 (10.0)
Epilepsy	8 (10.5)	7 (10.6)	1 (10.0)
Dyslipidemia	19 (25.0)	17 (25.8)	2 (20.0)
Cirrhosis	1 (1.3)	0 (0.0)	1 (10.0)
Systemic lupus erythematosus	5 (6.6)	5 (7.6)	0 (0.0)
HIV infection	18 (23.7)	18 (27.3)	0 (0.0)
Tuberculosis infection	15 (19.74)	15 (22.7)	0 (0.0)
Body temperature >39°C; *n* (%)	2 (2.6)	1 (1.5)	1 (10.0)
Heart rate >120 beats/min; *n* (%)	6 (7.9)	3 (4.6)	3 (30.0)
Final diagnosis; *n* (%)			
SJS	46 (60.5)	45 (68.2)	1 (10.0)
SJS–TEN overlap	15 (19.7)	13 (19.7)	2 (20.0)
TEN	15 (19.7)	8 (12.1)	7 (70.0)
Causality; *n* (%)			
Non–drug–related SJS/TEN	3 (4.0)	2 (3.0)	1 (10.0)
Drug–related SJS/TEN			
Single culprit drug	68 (89.5)	62 (93.9)	6 (60.0)
Multiple culprit drugs	4 (5.3)	2 (3.0)	2 (20.0)
Unidentified culprit drug(s)	1 (1.3)	0 (0.0)	1 (10.0)
Time interval form drug intake to onset of reaction, days; median (IQR)^‡^	13.5 (6.0, 24.0)	13.5 (6.0, 24.0)	13.5 (3.0, 42.0)
Late drug withdrawal; *n* (%)^‡^	45 (66.2)	41 (66.1)	4 (66.7)
Laboratory findings; *n* (%)			
BUN >10 mmol/L	11/74 (14.9)	6/64 (9.4)	5/10 (50.0)
Serum glucose >14 mmol/L	3/63 (4.8)	2/54 (3.7)	1/9 (11.1)
Serum HCO_3_ <20 mmol/L	22/71 (31.0)	18/62 (29.0)	4/9 (44.4)
Hb <10 g/dL	15/74 (20.3)	10/64 (15.6)	5/10 (50.0)
WBC count <1,000 or >20,000 cells/μL	3/75 (4.0)	2/65 (3.1)	1/10 (10.0)
Platelet count <150,000 or >450,000 cells/μL	14/75 (18.7)	11/65 (16.9)	3/10 (30.0)
Serum Na <125 or >145 mmol/L	0/72 (0.0)	0/63 (0.0)	0/9 (0.0)
Serum K <3 or >5 mmol/L	5/72 (6.9)	4/63 (6.4)	1/9 (11.1)
AST >40 U/L	37/69 (53.6)	31/61 (50.8)	6/8 (75.0)
ALT >40 U/L	44/69 (63.8)	38/61 (62.3)	6/8 (75.0)
Total bilirubin ≥68.4 mmol/L	6/69 (8.7)	4/61 (6.6)	2/8 (25.0)
Serum albumin <2 g/dL	4/70 (5.7)	1/61 (1.6)	3/9 (33.3)
Treatment; *n* (%)			
Systemic corticosteroids	72 (94.7)	64 (84.2)	8 (10.5)
Intravenous immunoglobulin	10 (13.2)	8 (10.5)	2 (2.6)

*The total number of patients was 76 unless specified.

^†^The number of patients with documented BMI was 56.

^‡^Data were from 68 patients having drug–related Stevens–Johnson syndrome and toxic epidermal necrolysis with single culprit drug.

ALT, alanine aminotransferase; AST, aspartate aminotransferase; BMI, body mass index; BUN, blood urea nitrogen; GFR, glomerular filtration rate; Hb, hemoglobin; HCO_3_, bicarbonate; HIV, human immunodeficiency virus; IQR, interquartile range; K, potassium; Na, sodium; SD, standard deviation; SJS, Stevens–Johnson syndrome; TEN, toxic epidermal necrolysis; WBC, white blood cell.

Drug was the major cause of disease, accounted in 73 (96.1%) patients, among whom 68 (89.5%) had a single culprit drug, 4 (5.3%) had multiple culprit drugs, and culprit drug could not be identified in 1 (1.3%). The culprit drugs are listed in Tables 2, 3. The most frequent culprit drugs were allopurinol (16.2%) and trimethoprim/sulfamethoxazole (16.2%), followed by phenytoin (13.2%), carbamazepine (7.4%), and rifampicin (5.9%).

**Table 2:** List of culprit drugs in patients with single culprit drug, categorized by drug notoriety (18, 21).

**Drug notoriety**	**Total (*n* = 68)**	**Deceased (*n* = 6)**
Strongly associated drugs		
Allopurinol	11	1
Trimethoprim/sulfamethoxazole	11	0
Phenytoin	9	2
Carbamazepine	5	0
Nevirapine	2	0
Piroxicam	2	0
Etoricoxib	2	0
Associated drugs		
Rifampicin	4	0
Levofloxacin	2	0
Amoxicillin	1	1
Ciprofloxacin	1	0
Suspected drugs		
Meropenem	2	1
Isoniazid	2	0
Celecoxib	2	0
Cloxacillin	1	0
Ofloxacin	1	0
Tetracycline	1	0
Omeprazole	1	0
Drugs not known to be associated		
Metronidazole	1	1
Vancomycin	1	0
Paracetamol	1	0
Ibuprofen	1	0
Mefenamic acid	1	0
Sulindac	1	0
Dapsone	1	0
Atezolizumab	1	0

**Table 3:** List of culprit drugs in patients with multiple culprit drugs.

**Case**	**ALDEN**	**Number**	**Culprit drugs**
**number**	**score**	**of drugs**	
1	5	2	Carbamazepine and phenytoin
2	3	2	Meropenem and trazodone
3	3	2	Ganciclovir and voriconazole
4	3	2	Capecitabine and exemestane

The results of univariate analysis of potential prognostic factors for mortality are shown in Tables 4, 5. Nine of them were significantly associated with death, including age, malignancy, CKD with baseline estimated GFR of either 30–89 or <30 mL/min per 1.73 m^2^, coronary artery disease, HR >120 beats/min, final diagnoses of SJS-TEN overlap and TEN, BUN >10 mmol/L, Hb <10 g/dL, and serum albumin <2 g/dL. They were included in the multivariate model selection, along with stroke, body temperature >39°C, and total bilirubin ≥68.4 mmol/L which yielded *p*-values of <0.2.

**Table 4:** Univariate analysis of potential prognostic factors for mortality in patients with Stevens–Johnson syndrome and toxic epidermal necrolysis.

**Factor**	**Risk ratio**	**95% CI**	***P*–value**
Age, per year	1.08	1.02–1.14	**0.006**
Female	0.60	0.18–1.96	0.397
BMI, per kg/m^2^	0.98	0.89–1.08	0.658
Medical comorbidity			
Malignancy	5.33	1.82–15.63	**0.002**
Chronic kidney disease	4.96	1.73–14.28	**0.003**
GFR ≥90 mL/min per 1.73 m^2^	1		
GFR 30–89 mL/min per 1.73 m^2^	4.77	1.58–14.36	**0.006**
GFR <30 mL/min per 1.73 m^2^	3.82	1.19–12.27	**0.024**
Coronary artery disease	4.5	1.39–14.61	**0.012**
Stroke	2.46	0.65–9.41	**0.187**
Hypertension	1.44	0.45–4.65	0.538
Diabetes mellitus	1.21	0.29–5.06	0.793
Gout	1.10	0.16–7.43	0.926
Epilepsy	0.94	0.14–6.52	0.954
Dyslipidemia	0.75	0.17–3.23	0.699
Body temperature >39°C	4.11	0.90–18.71	**0.067**
Heart rate >120 beats/min	5.00	1.72–14.50	**0.003**
Final diagnosis			
SJS	1		
SJS–TEN overlap	2.52	1.05–6.04	**0.038**
TEN	12.43	3.78–40.90	**0.000**
Causality			
Non–drug–related SJS/TEN	1		
Drug–related SJS/TEN	0.37	0.07–2.05	0.255
Laboratory findings			
BUN >10 mmol/L	5.73	1.98–16.55	**0.001**
Serum glucose >14 mmol/L	2.50	0.45–14.04	0.298
Serum HCO_3_ <20 mmol/L	1.78	0.53–6.00	0.351
Hb <10 g/dL	3.93	1.31–11.85	**0.015**
WBC count <1,000 or >20,000 cells/μL	2.67	0.48–14.79	0.262
Platelet count <150,000 or >450,000 cells/μL	1.87	0.55–6.33	0.316
Serum K <3 or >5 mmol/L	1.68	0.26–10.87	0.589
AST >40 U/L	2.59	0.56–11.97	0.222
ALT >40 U/L	1.70	0.37–7.82	0.493
Total bilirubin ≥68.4 mmol/L	3.50	0.90–13.69	**0.072**
Serum albumin <2 g/dL	8.25	3.19–21.33	**<0.001**

Variables with p–values < 0.2, indicated by bold font, were subsequently used in the multivariate model selection.

ALT, alanine aminotransferase; AST, aspartate aminotransferase; BMI, body mass index; BUN, blood urea nitrogen; CI, confidence interval; GFR, glomerular filtration rate; Hb, hemoglobin; HCO_3_, bicarbonate; K, potassium; SJS, Stevens–Johnson syndrome; TEN, toxic epidermal necrolysis; WBC, white blood cell.

**Table 5:** Univariate analysis of additional potential prognostic factors for mortality in patients having drug–related Stevens-Johnson syndrome and toxic epidermal necrolysis with single culprit drug.

	**Risk ratio**	**95% CI**	***P*-value**
Time interval form drug intake to onset of reaction	0.99	0.98–1.01	0.784
Late drug withdrawal	1.02	0.20–5.17	0.979
Drug notoriety			
Drug not known to be associated	1		
Suspected drug	0.80	0.05–11.75	0.868
Associated drug	1.00	0.07–14.65	1.000
Strongly associated drug	0.57	0.07–4.94	0.611

CI, confidence interval.

**Figure 2:**
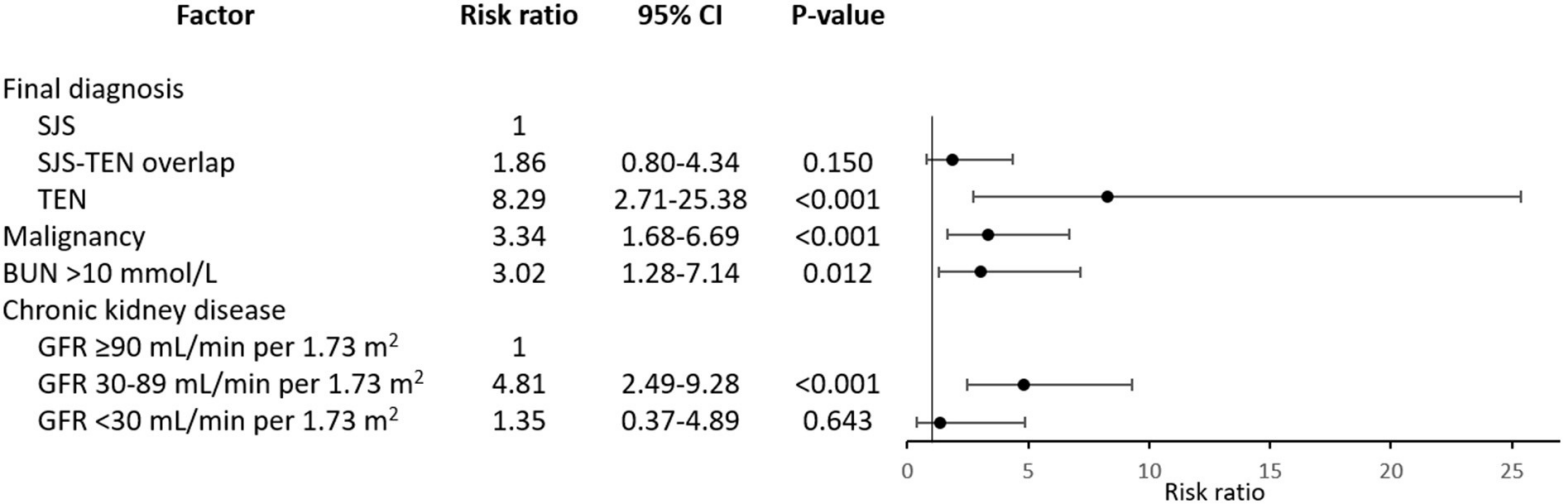
Independent prognostic factors for mortality in patients with Stevens-Johnson syndrome and toxic epidermal necrolysis. Risk ratios are adjusted for systemic therapy administered. BUN, blood urea nitrogen; CI, confidence interval; GFR, glomerular filtration rate; SJS, Stevens-Johnson syndrome; TEN, toxic epidermal necrolysis.

Subsequently, four variables were finally shown to be independent prognostic factors for mortality from the multivariate analysis adjusting for treatments with systemic corticosteroids and intravenous immunoglobulin, i.e., TEN, malignancy, BUN level >10 mmol/L at the presentation of SJS/TEN, and CKD with baseline estimated GFR of 30–89 mL/min per 1.73 m^2^ (Figure 2). Patients with TEN had an increased risk of death by approximately eight times relative to those with SJS (RR 8.29, 95% CI: 2.71–25.38). Both malignancy and BUN >10 mmol/L were associated with approximately three-fold increased mortality risk (RR 3.34, 95% CI: 1.68–6.69 and RR 3.02, 95% CI: 1.28–7.14, respectively). Meanwhile, CKD with baseline estimated GFR of 30–89 mL/min per 1.73 m^2^ showed almost a five-fold elevation in mortality risk in comparison to absence of CKD (RR 4.81, 95% CI: 2.49–9.28), independent of the BUN level at the presentation of SJS/TEN.

## Discussion

Our study was a 14-year retrospective cohort study aiming to identify additional prognostic factors for mortality of patients with SJS/TEN, with estimated mortality rates of SJS, SJS-TEN overlap, and TEN of 2.2, 13.3, and 46.7%, respectively. Using the ALDEN score, drug was considered to be the cause of epidermal necrolysis in the majority of cases, but the culprit drug could not be identified in 1.3% of them. The most frequent culprit drugs were allopurinol and trimethoprim/sulfamethoxazole, followed by phenytoin, carbamazepine, and rifampicin. These culprit drugs are considerably similar to those commonly found in previous studies from the United States (28), Europe (10), and other Asian countries (29), except for rifampicin, which could be due to the endemicity of tuberculosis in Thailand. However, there is some variation in the ranks among these most common culprit drugs between different regions, which might be explained by the difference in the patients' genetic background (30). A number of pharmacogenetic markers have been reported to be of high prevalence in the Thai population, i.e., HLA-B^*^58:01, HLA-A^*^33:03, and HLA-C^*^03:02 for allopurinol; HLA-C^*^08:01, HLA-C^*^14:02, and HLA-DRB1^*^12:02 for trimethoprim/sulfamethoxazole; and HLA-B^*^15:02 for carbamazepine and oxcarbazepine (31, 32).

Although a previous 10-year observational study of 113 patients with SJS/TEN suggested that withdrawal of the causative drug not later than the day of SJS/TEN definite sign occurrence was associated with the better prognosis (OR of 0.69 for earlier withdrawal by each day, 95% CI: 0.53–0.89) (14), the present study could not identify a significant association between late drug withdrawal and mortality. Likewise, significant associations of causality, drug notoriety, and time interval from drug intake to onset of reaction with mortality were not found in this study. Nonetheless, four independent prognostic factors for mortality after adjusting for the administered systemic therapy were identified, i.e., TEN, malignancy, high BUN level at the presentation of SJS/TEN, and pre-existing early-stage CKD. Even though late-stage CKD was also found to be significantly prognostic for death from the univariate analysis, the association became statistically non-significant as well as considerably weaker (as shown by the change in magnitude of the estimated RR from 3.82 to 1.35) in the multivariate analysis. The latter finding indicates confounding effects that other predictors in the model (i.e., systemic corticosteroids and intravenous immunoglobulin therapy, final diagnosis of SJS/TEN, malignancy, and BUN level) have on the association between late-stage CKD and death. Of note, the fact that all late-stage CKD patients had BUN level >10 mmol/L at the presentation of SJS/TEN strongly suggests a confounding effect from BUN level. However, similar confounding effects on the relationship between early-stage CKD and death were less likely, as shown by the minimal difference between the univariate and multivariate RRs. In other words, the association between early-stage CKD and death could not be explained by the elevated BUN level, and thus early-stage CKD can be considered predictive for mortality in the present study, independent of other significant factors including BUN level at the presentation of SJS/TEN.

Association between impaired renal function and mortality of SJS/TEN patients has been explored in previous studies, in the form of BUN levels, acute kidney injury, or states of renal insufficiency of unspecified chronicity (2, 12, 33–36). Significant association between impaired renal function and delayed clearance of plasma oxypurinol (i.e., the main metabolite of allopurinol) as well as the risk for severe cutaneous adverse reactions has been demonstrated in patients taking allopurinol. The resultant increases in plasma levels of oxypurinol and granulysin (i.e., a major cytotoxic and chemotactic factor in SJS/TEN) have also been found to significantly relate to mortality in allopurinol-induced SJS/TEN (37). In the present study, early-stage CKD has shown a moderate (38) association with the risk of mortality (RR 4.81, 95% CI: 2.49–9.28), regardless of the BUN level. To the best of our knowledge, CKD has never been reported as an independent prognostic factor for mortality in patients with SJS/TEN. These findings may suggest a raise in awareness while caring for SJS/TEN patients with CKD, even when the BUN level is not particularly high, which may subsequently lead to more prompt management in the event of rapid deterioration. However, our analysis should be considered exploratory, and therefore future studies are warranted to confirm these findings.

The present study also demonstrated statistically significant associations of other prognostic factors from the SCORTEN, including age and HR >120 beats/min, with the risk of death from the univariate analyses. Considering the seven prognostic factors from the SCORTEN, significant associations with death, at least from univariate analysis, were shown for TEN, malignancy, older age, HR >120 beats/min, and BUN >10 mmol/L, whereas positive but non-significant associations were observed for serum glucose >14 mmol/L and serum HCO_3_ <20 mmol/L. Overall, it can be inferred that the SCORTEN is an appropriate prognostic score for predicting mortality in Thai patients with SJS/TEN.

Apart from the risk factors from the SCORTEN, coronary artery disease, Hb <10 g/dL, and serum albumin <2 g/dL were significantly associated with mortality of patients with SJS/TEN from univariate analysis in the present study. Previous studies have reported associations between hypoalbuminemia and mortality in several settings including critical care, cardiovascular disease, renal disease, and thermal burns (24, 39). In SJS/TEN, a significant association between serum albumin <2.5 g/dL and mortality (OR 8.5, 95% CI: 2.43–29.75) has been shown in a previous study (23). As the functions of albumin include maintenance of plasma oncotic pressure, binding and transport properties, antioxidant effects, and anti-apoptotic effects, low levels of serum albumin could lead to third-space fluid loss, malnutrition, and abnormal immune responses, which could contribute to the increased mortality risk of patients with SJS/TEN.

There are limitations in our retrospective study. It was conducted in a single university-based hospital with a small sample size. The number of patients with CKD was also limited. Larger studies are needed to confirm our findings.

## Conclusions

CKD has been found to be a moderately strong, independent, prognostic factor for mortality of patients with SJS/TEN in the present study, along with BUN >10 mmol/L, TEN, and malignancy. Serum albumin <2 g/dL, coronary artery disease, and Hb <10 g/dL were also significantly associated with the risk of death from univariate analysis. Further studies are warranted to examine and confirm these associations.

## Data availability statement

The raw data supporting the conclusions of this article will be made available by the authors, without undue reservation.

## Ethics statement

The studies involving human participants were reviewed and approved by Human Research Ethics Committee, Faculty of Medicine Ramathibodi Hospital, Mahidol University. Written informed consent for participation was not required for this study in accordance with the national legislation and the institutional requirements.

## Author contributions

PR, PJ, and KT conceptualized and designed the study. PPa, PPi, and PJ collected the data. PPa, PJ, and KT analyzed the data. PJ and PPa drafted the manuscript. PR and KT made critical revisions of the manuscript. All authors contributed to the article and approved the submitted version.

## Conflict of interest

The authors declare that the research was conducted in the absence of any commercial or financial relationships that could be construed as a potential conflict of interest.

## Publisher's note

All claims expressed in this article are solely those of the authors and do not necessarily represent those of their affiliated organizations, or those of the publisher, the editors and the reviewers. Any product that may be evaluated in this article, or claim that may be made by its manufacturer, is not guaranteed or endorsed by the publisher.

## References

[1] Yang MS Lee JY Kim J Kim GW Kim BK Kim JY. Incidence of Stevens-Johnson syndrome and toxic epidermal necrolysis: a nationwide population-based study using national Health Insurance Database in Korea. PLoS ONE. (2016) 11:e0165933. 10.1371/journal.pone.0165933 27835661 PMC5106005

[2] Hsu DY Brieva J Silverberg NB Silverberg JI. Morbidity and mortality of Stevens-Johnson syndrome and toxic epidermal necrolysis in United States Adults. J Invest Dermatol. (2016) 136:1387–97. 10.1016/j.jid.2016.03.023 27039263

[3] Schöpf E Stühmer A Rzany B Victor N Zentgraf R Kapp JF. toxic epidermal necrolysis and Stevens-Johnson syndrome. An epidemiologic study from West Germany. Arch Dermatol. (1991) 127:839–42. 10.1001/archderm.1991.01680050083008 2036029

[4] Papp A Sikora S Evans M Song D Kirchhof M Miliszewski M. Treatment of toxic epidermal necrolysis by a multidisciplinary team. A review of literature and treatment results. Burns. (2018) 44:807–15. 10.1016/j.burns.2017.10.022 29627131

[5] Dodiuk-Gad RP Chung WH Valeyrie-Allanore L Shear NH. Stevens-Johnson syndrome and toxic epidermal necrolysis: an update. Am J Clin Dermatol. (2015) 16:475–93. 10.1007/s40257-015-0158-0 26481651

[6] Chantaphakul H Sanon T Klaewsongkram J. clinical characteristics and treatment outcome of Stevens-Johnson syndrome and toxic epidermal necrolysis. Exp Ther Med. (2015) 10:519–24. 10.3892/etm.2015.2549 26622347 PMC4509461

[7] Assier H Bastuji-Garin S Revuz J Roujeau JC. Erythema multiforme with mucous membrane involvement and Stevens-Johnson syndrome are clinically different disorders with distinct causes. Arch Dermatol. (1995) 131:539–43. 10.1001/archderm.1995.01690170041005 7741539

[8] Duong TA Valeyrie-Allanore L Wolkenstein P Chosidow O. Severe cutaneous adverse reactions to drugs. Lancet. (2017) 390:1996–2011. 10.1016/S0140-6736(16)30378-6 28476287

[9] Mockenhaupt M. The current understanding of Stevens-Johnson syndrome and toxic epidermal necrolysis. Expert Rev Clin Immunol. (2011) 7:803–13. 10.1586/eci.11.66 22014021

[10] Sassolas B Haddad C Mockenhaupt M Dunant A Liss Y Bork K. Alden, an algorithm for assessment of drug causality in Stevens-Johnson syndrome and toxic epidermal necrolysis: comparison with case-control analysis. Clin Pharmacol Ther. (2010) 88:60–8. 10.1038/clpt.2009.252 20375998

[11] Schwartz RA McDonough PH Lee BW. Toxic epidermal necrolysis: part i. introduction, history, classification, clinical features, systemic manifestations, etiology, and immunopathogenesis. J Am Acad Dermatol. (2013) 69:173.e1–13. 10.1016/j.jaad.2013.05.003 23866878

[12] Bastuji-Garin S Fouchard N Bertocchi M Roujeau JC Revuz J Wolkenstein P. Scorten: a severity-of-illness score for toxic epidermal necrolysis. J Invest Dermatol. (2000) 115:149–53. 10.1046/j.1523-1747.2000.00061.x 10951229

[13] Torres-Navarro I Briz-Redón Á Botella-Estrada R. Accuracy of scorten to predict the prognosis of Stevens-Johnson syndrome/toxic epidermal necrolysis: a systematic review and meta-analysis. J Eur Acad Dermatol Venereol. (2019). 10.1111/jdv.16137 31912590

[14] Garcia-Doval I LeCleach L Bocquet H Otero XL Roujeau JC. Toxic epidermal necrolysis and Stevens-Johnson syndrome: does early withdrawal of causative drugs decrease the risk of death? Arch Dermatol. (2000) 136:323–7. 10.1001/archderm.136.3.323 10724193

[15] Quinn AM Brown K Bonish BK Curry J Gordon KB Sinacore J. Uncovering histologic criteria with prognostic significance in toxic epidermal necrolysis. Arch Dermatol. (2005) 141:683–7. 10.1001/archderm.141.6.683 15967913

[16] Davis RL Gallagher MA Asgari MM Eide MJ Margolis DJ Macy E. Identification of Stevens-Johnson syndrome and toxic epidermal necrolysis in electronic health record databases. Pharmacoepidemiol Drug Saf. (2015) 24:684–92. 10.1002/pds.3778 25914229 PMC7105169

[17] Bastuji-Garin S Rzany B Stern RS Shear NH Naldi L Roujeau JC. Clinical classification of cases of toxic epidermal necrolysis, Stevens-Johnson syndrome, and erythema multiforme. Arch Dermatol. (1993) 129:92–6. 10.1001/archderm.1993.01680220104023 8420497

[18] Roujeau JC Kelly JP Naldi L Rzany B Stern RS Anderson T. Medication use and the risk of Stevens-Johnson syndrome or toxic epidermal necrolysis. N Engl J Med. (1995) 333:1600–7. 10.1056/NEJM199512143332404 7477195

[19] Yang MS Lee JY Kim J Kim GW Kim BK Kim JY. Searching for the culprit drugs for Stevens-Johnson syndrome and toxic epidermal necrolysis from a nationwide claim database in Korea. J Allergy Clin Immunol Pract. (2020) 8:690-5.e2. 10.1016/j.jaip.2019.09.032 31614216

[20] Kelly JP Auquier A Rzany B Naldi L Bastuji-Garin S Correia O. An international collaborative case-control study of Severe Cutaneous Adverse Reactions (Scar). Design and methods. J Clin Epidemiol. (1995) 48:1099–108. 10.1016/0895-4356(95)00004-N 7636511

[21] The RegiSCAR Project. Drug Notoriety for Alden. (2017). Available online at: http://www.regiscar.org/pdf/Drug%20Notoriety%202015.%20revised%20may%202017.xls (accessed May 18, 2021).

[22] Levin A Stevens PE. Summary of KDIGO 2012 CKD guideline: behind the scenes, need for guidance, and a framework for moving forward. Kidney Int. (2014) 85:49–61. 10.1038/ki.2013.444 24284513

[23] Kannenberg SM Jordaan HF Koegelenberg CF Von Groote-Bidlingmaier F Visser WI. Toxic epidermal necrolysis and Stevens-Johnson syndrome in South Africa: A 3-year prospective study. QJM. (2012) 105:839–46. 10.1093/qjmed/hcs078 22543685

[24] Aguayo-Becerra OA Torres-Garibay C Macías-Amezcua MD Fuentes-Orozco C Chávez-Tostado Mde G Andalón-Dueñas E. Serum albumin level as a risk factor for mortality in burn patients. Clinics. (2013) 68:940–5. 10.6061/clinics/2013(07)09 23917657 PMC3714858

[25] Guégan S Bastuji-Garin S Poszepczynska-Guigné E Roujeau JC Revuz J. Performance of the scorten during the first five days of hospitalization to predict the prognosis of epidermal necrolysis. J Invest Dermatol. (2006) 126:272–6. 10.1038/sj.jid.5700068 16374461

[26] Norton EC Miller MM Kleinman LC. Computing adjusted risk ratios and risk differences in stata. Stata J. (2013) 13:492–509. 10.1177/1536867X130130030434855941

[27] Knol MJ Le Cessie S Algra A Vandenbroucke JP Groenwold RH. Overestimation of risk ratios by odds ratios in trials and cohort studies: alternatives to logistic regression. CMAJ. (2012) 184:895–9. 10.1503/cmaj.101715 22158397 PMC3348192

[28] Micheletti RG Chiesa-Fuxench Z Noe MH Stephen S Aleshin M Agarwal A. Stevens-Johnson syndrome/toxic epidermal necrolysis: a multicenter retrospective study of 377 adult patients from the United states. J Invest Dermatol. (2018) 138:2315–21. 10.1016/j.jid.2018.04.027 29758282

[29] Wang YH Chen CB Tassaneeyakul W Saito Y Aihara M Choon SE. The medication risk of Stevens-Johnson syndrome and toxic epidermal necrolysis in Asians: the major drug causality and comparison with the US Fda Label. Clin Pharmacol Ther. (2019) 105:112–20. 10.1002/cpt.1071 29569740

[30] Chang CJ Chen CB Hung SI Ji C Chung WH. Pharmacogenetic testing for prevention of severe cutaneous adverse drug reactions. Front Pharmacol. (2020) 11:969. 10.3389/fphar.2020.00969 32714190 PMC7346738

[31] Nakkam N Konyoung P Kanjanawart S Saksit N Kongpan T Khaeso K. Hla Pharmacogenetic markers of drug hypersensitivity in a Thai population. Front Genet. (2018) 9:277. 10.3389/fgene.2018.00277 30127801 PMC6087736

[32] Su SC Hung SI Fan WL Dao RL Chung WH. Severe cutaneous adverse reactions: the pharmacogenomics from research to clinical implementation. Int J Mol Sci. (2016) 17:1890. 10.3390/ijms17111890 27854302 PMC5133889

[33] Lee TH Lee CC Ng CY Chang MY Chang SW Fan PC. The influence of acute kidney injury on the outcome of Stevens-Johnson syndrome and toxic epidermal necrolysis: the prognostic value of Kdigo staging. PLoS ONE. (2018) 13:e0203642. 10.1371/journal.pone.0203642 30192870 PMC6128626

[34] Noe MH Rosenbach M Hubbard RA Mostaghimi A Cardones AR Chen JK. Development and validation of a risk prediction model for in-hospital mortality among patients with Stevens-Johnson syndrome/toxic epidermal necrolysis-Abcd-10. JAMA Dermatol. (2019) 155:448–54. 10.1001/jamadermatol.2018.5605 30840032 PMC6459085

[35] Wang L Mei XL. Retrospective analysis of Stevens-Johnson syndrome and toxic epidermal necrolysis in 88 Chinese patients. Chin Med J. (2017) 130:1062–8. 10.4103/0366-6999.204929 28469101 PMC5421176

[36] Traikia C Hua C Le Cleach L de Prost N Hemery F Bettuzzi T. Individual- and hospital-level factors associated with epidermal necrolysis mortality: a nationwide multilevel study, France, 2012-2016. Br J Dermatol. (2020) 182:900–6. 10.1111/bjd.18294 31260078

[37] Chung WH Chang WC Stocker SL Juo CG Graham GG Lee MH. Insights into the poor prognosis of allopurinol-induced severe cutaneous adverse reactions: the impact of renal insufficiency, high plasma levels of oxypurinol and granulysin. Ann Rheum Dis. (2015) 74:2157–64. 10.1136/annrheumdis-2014-205577 25115449

[38] Olivier J May WL Bell ML. Relative effect sizes for measures of risk. Commun Stat. (2017) 46:6774–81. 10.1080/03610926.2015.1134575

[39] Goldwasser P Feldman J. Association of serum albumin and mortality risk. J Clin Epidemiol. (1997) 50:693–703. 10.1016/S0895-4356(97)00015-2 9250267

